# Characterization of a ferroptosis-related gene signature predicting survival and immunotherapeutic response in lung adenocarcinoma

**DOI:** 10.18632/aging.206110

**Published:** 2024-09-18

**Authors:** Chuan Zhang, Yingying Su, Hongrui Wang, Dan Dang, Xin Huang, Shuyou Shi, Yue Shi, Peng Zhang, Ming Yang

**Affiliations:** 1 Department of Pediatric Surgery, The First Hospital of Jilin University, Changchun, China; 2 Department of Anatomy, College of Basic Medical Sciences, Jilin University, Jilin, China; 3 Department of Molecular Biology, College of Basic Medical Sciences, Jilin University, Changchun, China; 4 Department of Neonatology, The First Hospital of Jilin University, Changchun, China; 5 Department of Microbiology and Immunology, Changchun University of Chinese Medicine, Changchun, China; 6 Department of Thoracic Surgery, The First Hospital of Jilin University, Changchun, China

**Keywords:** lung adenocarcinoma, ferroptosis, prognosis, drug response, gene signature

## Abstract

Lung cancer remains the leading cause of cancer-related death worldwide, and drug resistance represents the main obstacle responsible for the poor mortality and prognosis. Here, to identify a novel gene signature for predicting survival and drug response, we jointly investigated RNA sequencing data of lung adenocarcinoma patients from TCGA and GEO databases, and identified a ferroptosis-related gene signature. The signature was validated in the validation set and two external cohorts. The high-risk group had a reduced survival than the low-risk group (*P* < 0.05). Moreover, the established gene signature was associated with tumor mutation burden, microsatellite instability, and response to immune checkpoint blockade. In addition, four candidate oncogenes (*RRM2*, *SLC2A1*, *DDIT4*, and *VDAC2*) were identified to be candidate oncogenes using *in silico* and wet experiments, which could serve as potential therapeutic targets. Collectively, this study developed a novel ferroptosis-related gene signature for predicting prognosis and drug response, and identified four candidate oncogenes for lung adenocarcinoma.

## INTRODUCTION

Lung adenocarcinoma accounts for approximately 85% of pulmonary cancer [1]. The prognosis of advanced lung cancer is unsatisfactory owing to its heterogeneity [2, 3], with a five-year survival rate ranging from 4% to 17% [4]. Accurate prediction of patient survival is beneficial to clinical decision-making; nevertheless, there are limited tools to forecast the prognosis of lung adenocarcinoma [5]. Recently, integration of multiple biomarkers into a single signature has emerged to be an effective approach [6]. Therefore, the present study devoted to the development of a gene signature using RNA sequencing data to predict the survival of lung adenocarcinoma.

Despite remarkable advances in tumor molecular targeted therapy and immunotherapy, the survival of lung adenocarcinoma remains undesirable due to the drug resistance. For instance, EGFR-TKIs can enhance progression-free survival compared to conventional chemotherapy in lung adenocarcinoma patients, while most patients eventually developed resistance to EGFR-TKIs [7]. Likewise, PD-1/PD-L1 blockade has emerged to be a promising immunotherapy, but still is hampered by drug resistance [8]. Collectively, therapeutic resistance is a main challenge for cancer therapy, and risk stratification of cancer patients with different drug sensitiveness can help reduce therapeutic resistance. Notably, the establishment of a gene signature seems to be an effective tool to predict drug resistance in cancer treatment.

Ferroptosis exerts a pivotal role in various types of cancer, including lung cancer, renal cancer, pancreatic cancer, and diffuse large B-cell lymphoma [9–11]. Inhibition of iron-sulfur cluster biosynthetic enzyme NFS1 can trigger ferroptosis and suppress tumor cell growth [12], implying that ferroptosis could be a promising approach for cancer therapy [13]. Accordingly, ferroptosis-related genes could be used to therapeutic targets for cancer treatment.

Here, we identified a ferroptosis-related seven-gene signature by jointly interrogating the RNA sequencing data of lung adenocarcinoma patients from three independent datasets. Moreover, seven genes were further investigated using lung adenocarcinoma samples. The findings of the study are expected to provide more clues for the pathogenesis and prognosis prediction of lung adenocarcinomas.

## RESULTS

### Establishment of the prognostic ferroptosis-related gene signature

**Figure 1:**
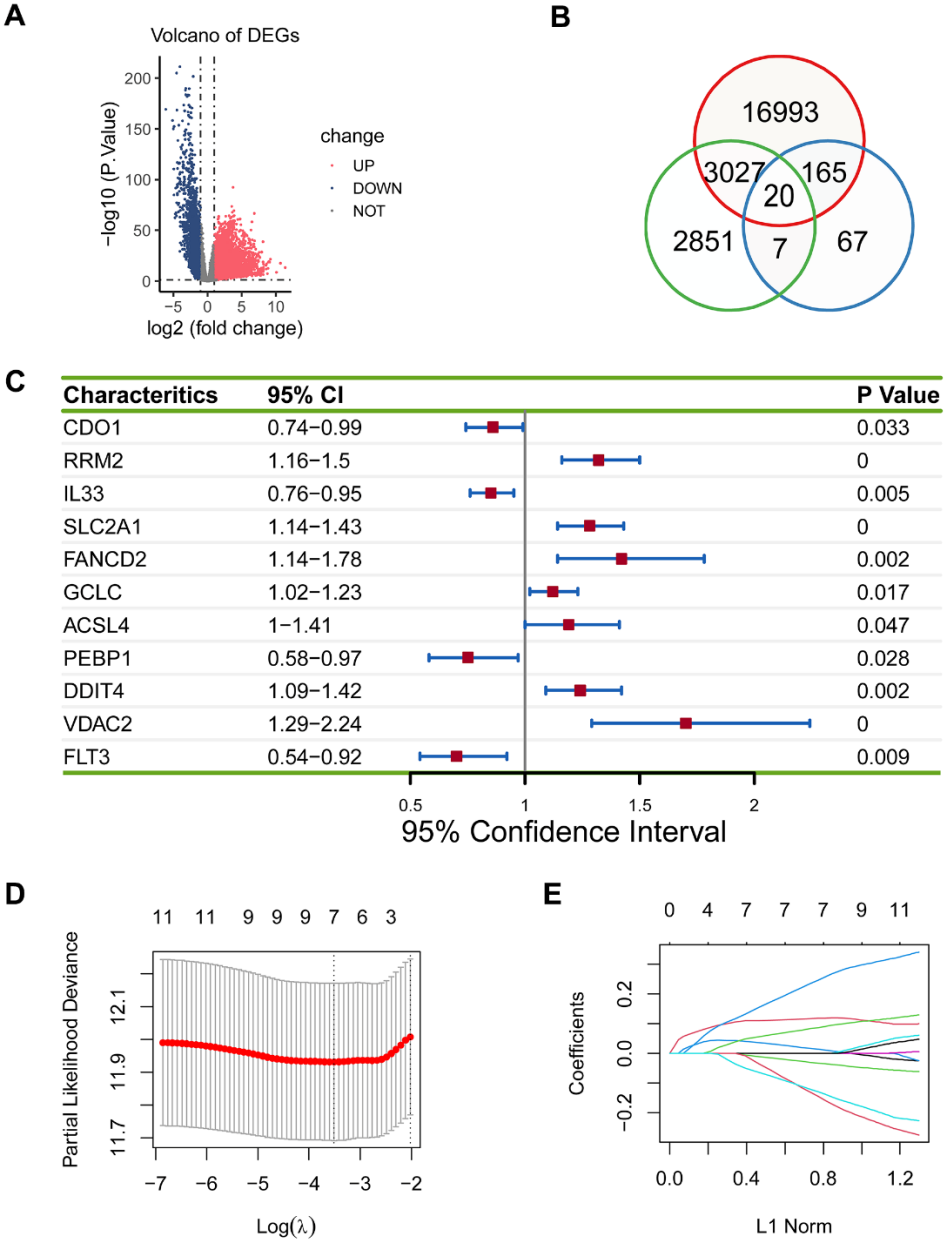
**Establishment of the prognostic ferroptosis-related gene signature.** (**A**) Volcano plot showed differentially expressed genes in lung adenocarcinoma. Blue dots represent downregulated genes, red dots represent upregulated genes, and grey dots represent unchanged genes. (**B**) There were 20 overlapping genes among 20205 DEGs, 5905 prognostically relevant genes, and 259 ferroptosis genes. (**C**) 11 eligible genes were obtained in the univariate Cox regression model. (**D**, **E**) Seven genes (*RRM2, IL33, SLC2A1, PEBP1, DDIT4, VDAC2*, and *FLT3*) were acquired in LASSO regression model.

To establish the ferroptosis-related gene signature in lung adenocarcinoma, we needed to identify the appropriate genes for the gene signature. We first performed a differential expression analysis between 526 tumor samples and 59 control samples, and obtained 20205 DEGs (Figure 1A). Then we performed a log-rank test for 52113 genes of lung adenocarcinoma patients from the TCGA cohort, and acquired 5905 prognostically relevant genes (*P* < 0.05). 259 ferroptosis genes were available on FerrDb. Finally, we intersected 20205 DEGs, 5905 prognostically relevant genes, and 259 ferroptosis genes, and gained 20 genes (Figure 1B).

The 20 genes were subjected to the univariate Cox regression model, and 11 eligible genes were obtained (*P* < 0.05; Figure 1C), which were then analyzed using LASSO. Seven genes (*RRM2, IL33, SLC2A1, PEBP1, DDIT4, VDAC2*, and *FLT3*) were acquired (*P* < 0.05; Figure 1D, 1E).

### Validation of the predictive ability of the ferroptosis-related gene signature

**Figure 2:**
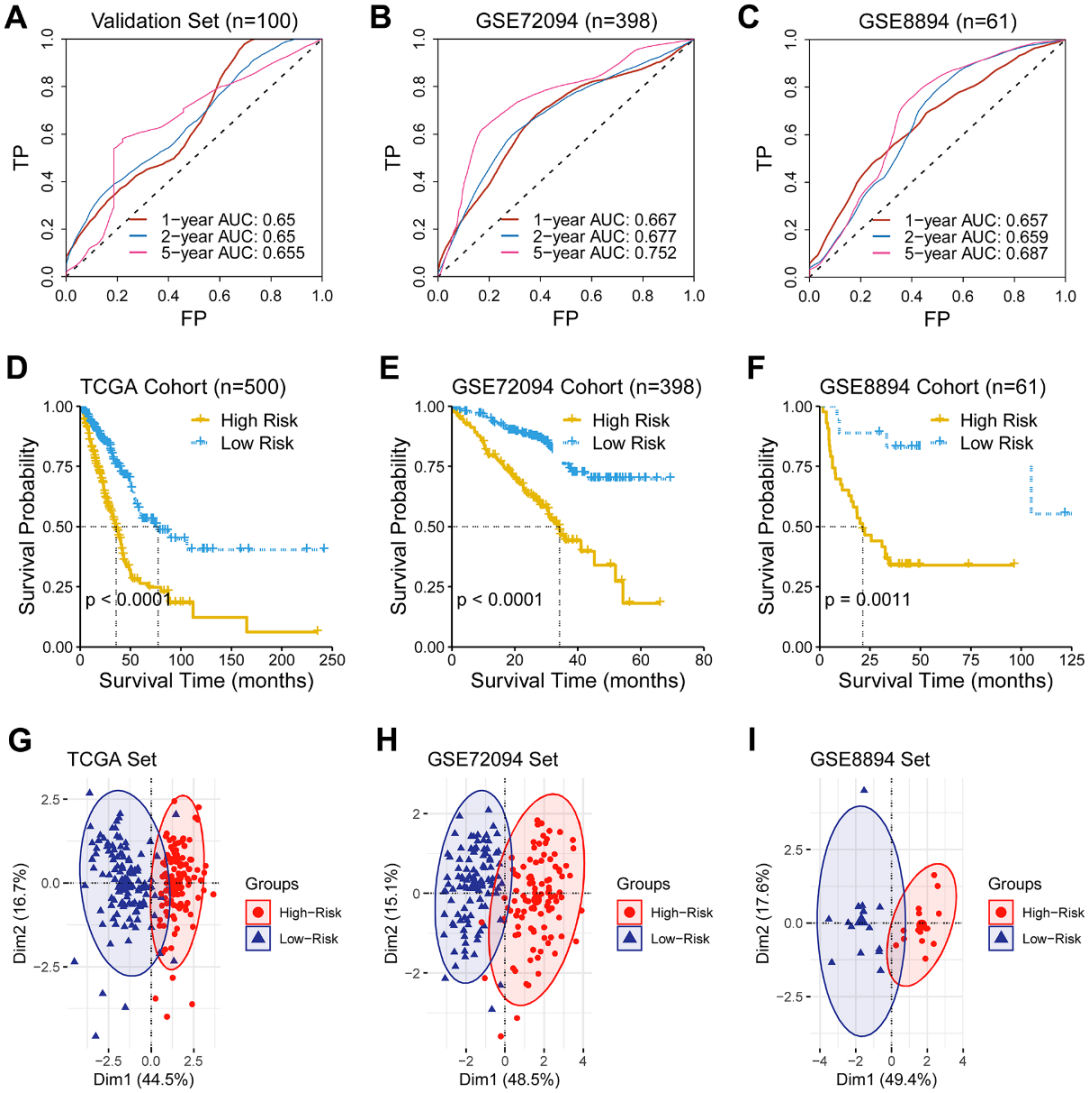
**Validation of the predictive ability of the ferroptosis-related gene signature.** (**A**–**C**) A receiver operating characteristic (ROC) curve was performed and area under the curve (AUC) was calculated in the validation set and two external test sets (GSE72094 and GSE8894). (**D**–**F**) Survival analysis was performed in the TCGA cohort, the GSE72094 cohort, and the GSE8894, respectively. (**G**–**I**) Principal component analysis of genes consisting the prognostic ferroptosis-related signature revealed a distinct expression pattern between the low- and the high-risk groups in dimensionality.

AUC for five-year overall survival was 0.655, 0.752, and 0.687 in the validation set, the GSE72094 cohort, and the GSE8894, respectively (Figure 2A–2C). Notably, the low-risk group demonstrated an improved survival than the high-risk group in the TCGA cohort and two other external cohorts (log-rank test, *P* < 0.05; Figure 2D, 2F). Similarly, PCA results revealed a different mode between the low- and the high-risk groups (Figure 2G–2I).

### Comparison of the predicting capacity between the signature and TNM staging

**Figure 3:**
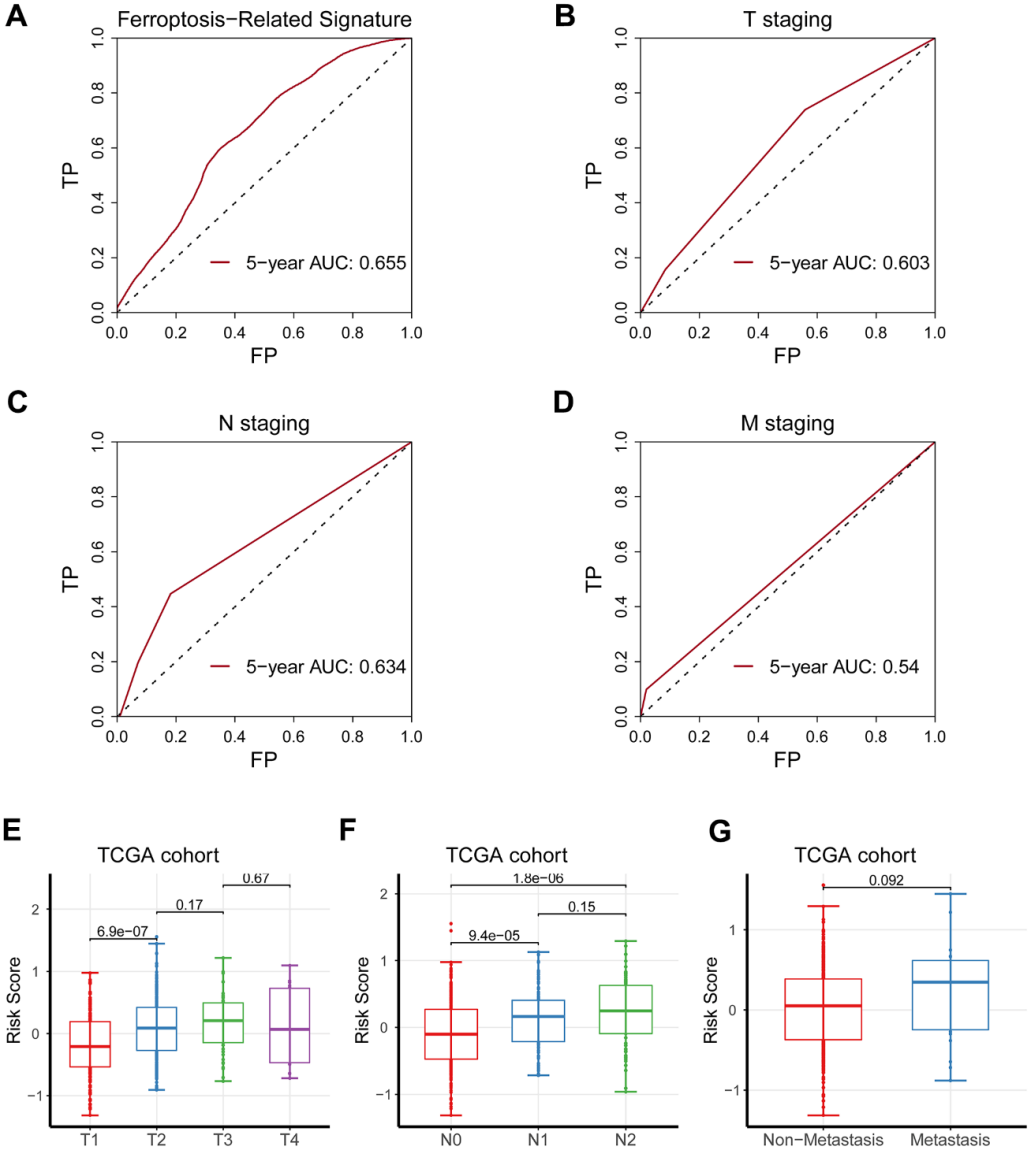
**Comparison of the predicting capacity between the signature and TNM staging.** (**A**) Five-year AUC value for the ferroptosis-related gene signature was 0.655. (**B**) Five-year AUC value for T staging was 0.603. (**C**) Five-year AUC value for N staging was 0.634. (**D**) Five-year AUC value for M staging was 0.540. (**E**–**G**) The risk score was significantly augmented as TNM staging increased.

The signature displayed a better forecasting ability than TNM staging, having an AUC value of 0.655 vs. 0.603, 0.634, and 0.540 (Figure 3A–3D). Moreover, the risk score based on the signature was remarkedly elevated as the TNM staging increased (Figure 3E–3G).

### Investigation of the signature-related biological function

**Figure 4:**
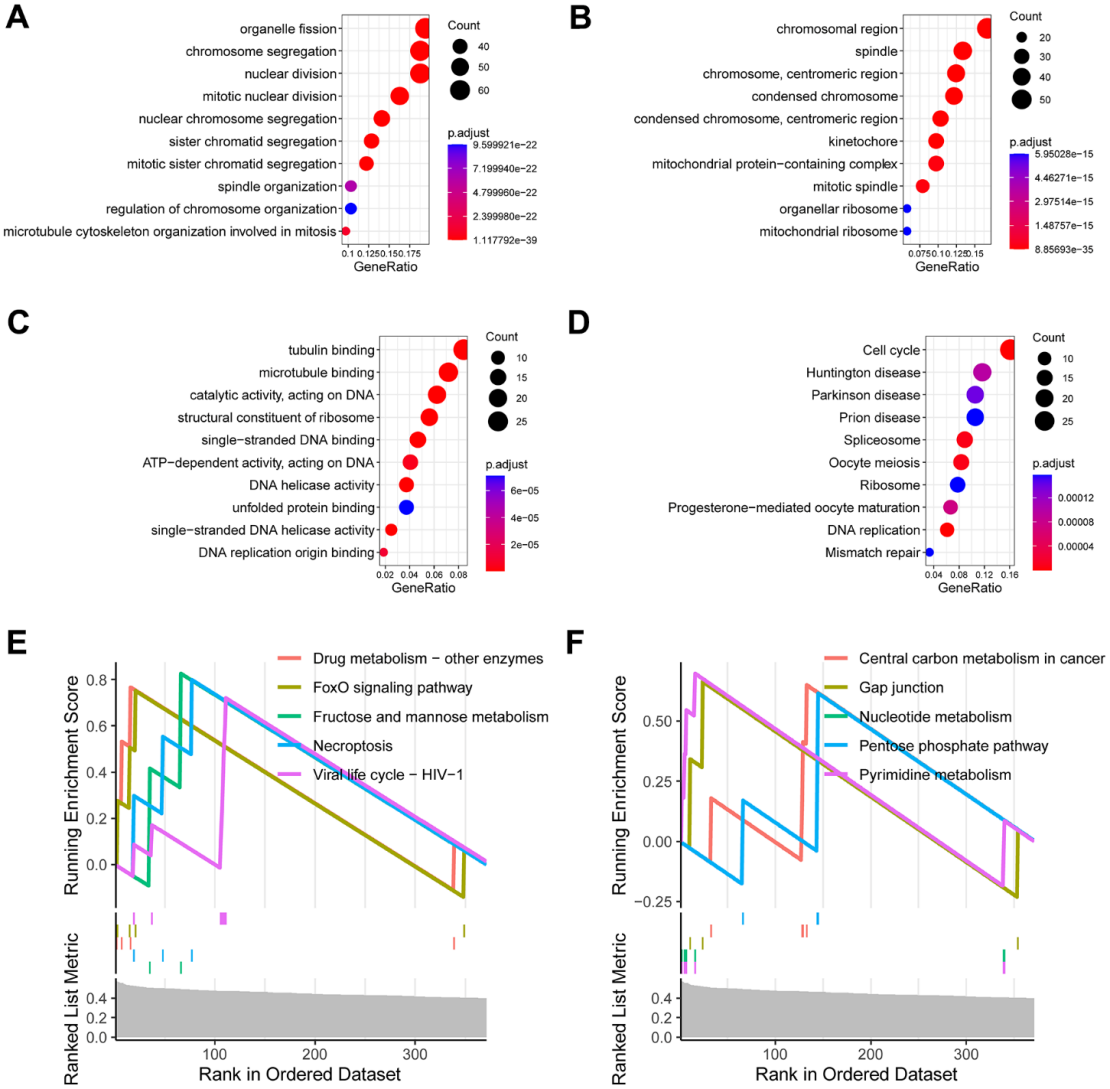
**Investigation of the signature-related biological function.** (**A**) Enriched terms of biological process in gene ontology analysis. (**B**) Enriched terms of cell component in gene ontology analysis. (**C**) Enriched terms of molecular function in gene ontology analysis. (**D**) Enriched terms of KEGG pathway in gene ontology analysis. (**E**, **F**) Enriched terms of KEGG pathway in gene set enrichment analysis (GSEA).

We further investigated of the signature-related biological function. A total of 361 signature-related genes (*P* < 0.01, *R* > 0.4) [14–16] were analyzed for their biological functions. The results showed that organelle fission, chromosomal region, tubulin binding, as well as cell cycle were significantly enriched (Figure 4A–4D). Similarly, the GSEA analysis observed drug metabolism, FoxO signaling pathway, necroptosis, and central carbon metabolism in cancer (Figure 4E, 4F).

### Profiling of tumor immune microenvironment of lung adenocarcinoma

**Figure 5:**
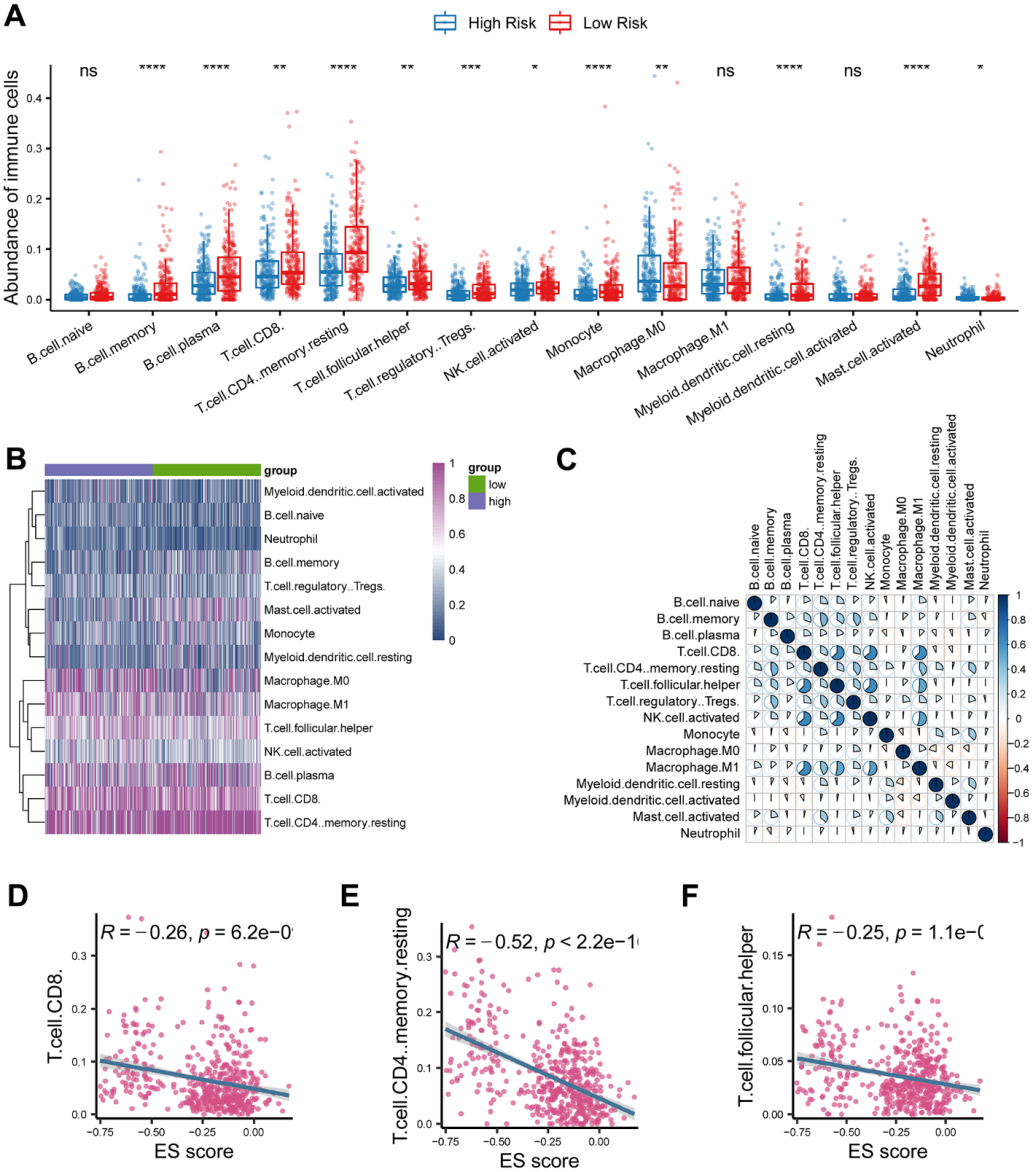
**Investigation of tumor immune microenvironment of lung adenocarcinoma.** (**A**) Comparison of tumor-infiltrating immune cells between cancerous and normal tissue. (**B**) Heatmap analysis of tumor-infiltrating immune cells in lung adenocarcinoma. (**C**) Correlations analysis of tumor-infiltrating immune cells in lung adenocarcinoma. (**D**–**F**) The risk score based on the ferroptosis-related gene signature was negatively correlated with CD8 T cell, CD4 T cell, and follicular helper T cell. * represents *P* < 0.05, ** represents *P* < 0.01, *** represents *P* < 0.001.

We found that CD8+ T cells and CD4+ T cells were significantly decreased in the high-risk group than in the low-risk group (Figure 5A). Heatmap also found the immune cells were distinctively distributed between the two groups (Figure 5B). Additionally, CD8+ T cell was positively correlated with follicular helper T cell, activated NK cell, and M1 macrophage (Figure 5C). Consistently, the risk score was adversely associated with CD8+ T cells, follicular helper T cells and CD4+ T cells (Figure 5D; *P* < 0.05).

### Profiling of somatic nucleotide variation in lung adenocarcinoma

**Figure 6:**
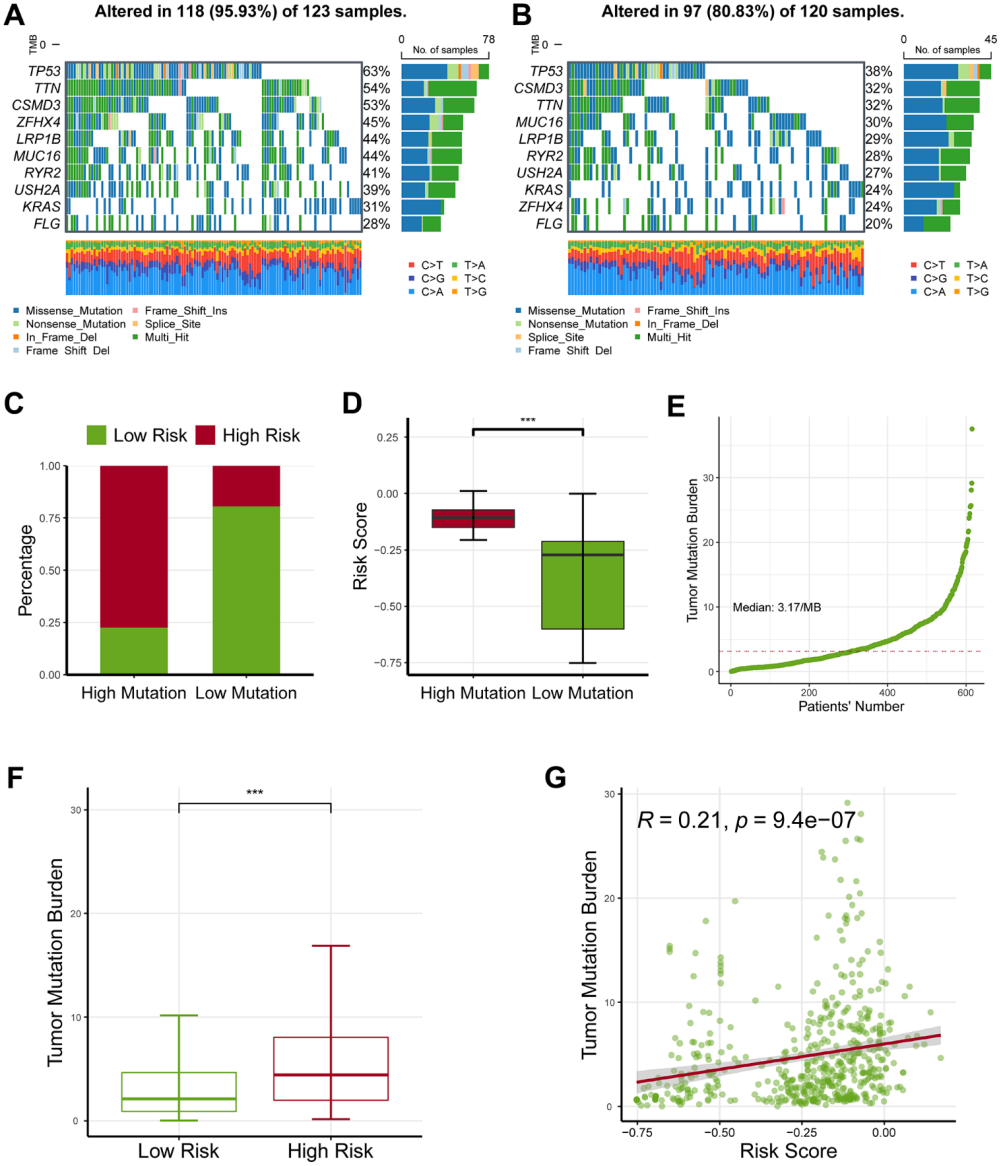
**Profiling of somatic nucleotide variation for lung adenocarcinoma patients.** (**A**) The waterfall plot showed that the high-risk group had a nucleotide variation rate of 95.93%. (**B**) The waterfall plot showed that the low-risk group had a nucleotide variation rate of 80.83%. (**C**) The bar plot showed that the risk score was critically increased in the high-mutation group than in the low-mutation group. (**D**) The box plot demonstrated that the risk score was critically increased in the high-mutation group than in the low-mutation group. (**E**) Tumor mutation burden (TMB) for lung adenocarcinoma patients. (**F**) The high-risk group had an increased TMB level as compared with the low-risk group. (**G**) The TMB levels were also positively correlated with the risk score. * represents *P* < 0.05, ** represents *P* < 0.01, *** represents *P* < 0.001.

The waterfall map revealed that the high-risk group had a more frequent nucleotide variation rate than the low-risk group (95.93% vs. 80.83%, Figure 6A, 6B). The bar chart found the risk was critically higher in the high-variation group than in the low-variation group (*P* < 0.05; Figure 6C), and box chart found the risk was critically augmented in the high- variation group than in the low-variation group (Figure 6D).

Moreover, we estimated the levels of tumor mutation burden (TMB) for lung adenocarcinoma patients (Figure 6E), and observed that the high-risk group had an increased TMB level as compared with the low-risk group (*P* < 0.05; Figure 6F). Consistently, TMB value was linked to the risk (Figure 6G). Collectively, these findings revealed that the ferroptosis-related gene signature might serve as an indicator of response to ICB.

### Impact of the ferroptosis-related signature on immunotherapeutic efficacy

**Figure 7:**
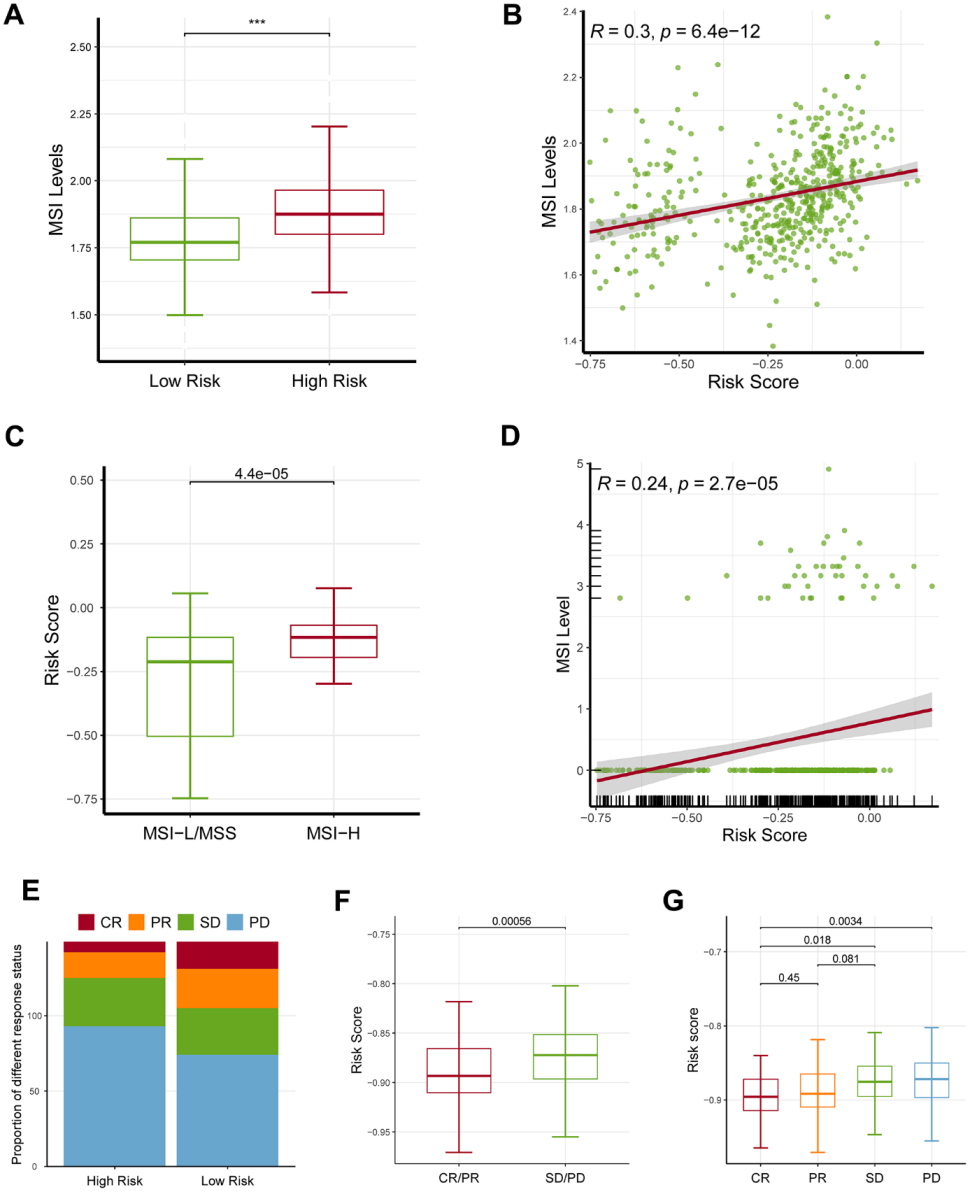
**Effects of the ferroptosis-related gene signature on response to ICB.** (**A**) The high-risk patients had a higher MSI level than the low-risk patients based on ssGSEA approach. (**B**) The risk score was positively correlated with MSI level based on ssGSEA approach. (**C**) MSI-H patients had a higher risk score level than MSI-L/MSS patients based on UCSCXenaShiny approach. (**D**) The risk score was positively correlated with MSI level based on UCSCXenaShiny approach. (**E**) The high-risk group had a higher proportion of stable disease (SD) and progressive disease (PD) than the low-risk group. (**F**, **G**) The SD/PD patients had an increased risk score than the CR/PR group. MSI: microsatellite instability; MSS: microsatellite stability, MSI-L: microsatellite instability-low, MSI-H: microsatellite instability-high, SD: stable disease, PD: progressive disease, CR: complete response, PR: partial response. * represents *P* < 0.05, ** represents *P* < 0.01, *** represents *P* < 0.001.

Microsatellite instability (MSI) level was also considered to be a predictor of response to ICB, therefore we next investigated the relationship between MSI and the signature. To increase the robustness of the results, we estimated the MSI levels using two independent bioinformatics methods: ssGSEA and the USCSXenaShiny. Consistently, both of the results of two independent approaches revealed that the high-risk patients owned an increased MSI level, and a positive relationship was demonstrated (Figure 7A–7D). Remarkably, the high-risk population showed a worse therapeutic effect than the low-risk group (Figure 7E). In accordance with that, the SD/PD patients had an increased risk score than the CR/PR group (Figure 7F, 7G).

### Identification of the hub genes in lung adenocarcinoma

**Figure 8:**
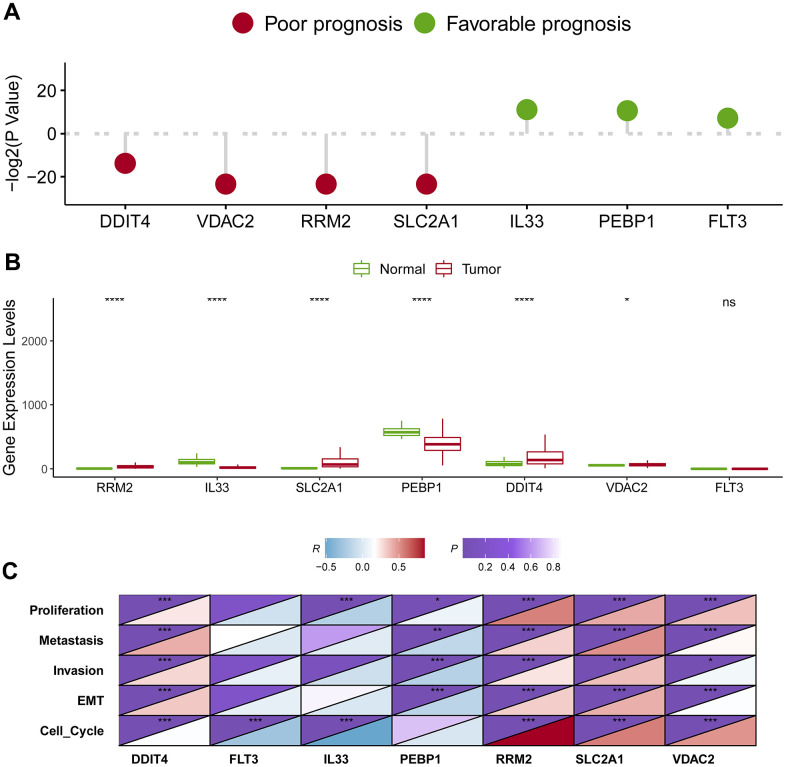
**Identification of the hub genes in lung adenocarcinoma.** (**A**) Survival analysis of seven candidate hub genes in lung adenocarcinoma. (**B**) Expression level analysis of seven candidate hub genes in lung adenocarcinoma. (**C**) The association of the hub gene expression levels and the levels of proliferation, invasion, metastasis, cell cycle and EMT in lung adenocarcinoma. * represents *P* < 0.05, ** represents *P* < 0.01, *** represents *P* < 0.001.

Survival analysis showed that four genes were linked with unwanted survival (*RRM2*, *SLC2A1*, *DDIT4*, and *VDAC2*; *P* < 0.05; Figure 8A), while three genes were associated with improved prognosis (*PEBP1*, *IL33*, and *FLT3*; *P* < 0.05; Figure 8A). Consistently, the four genes with poor survival were upregulated in cancerous samples, while the two genes with better survival (*PEBP1* and *IL33*) were downregulated in tumor samples (Figure 8B). Notably, *FLT3* expression was not significantly changed between tumor and normal samples. We noticed that *FLT3* mRNA expression was comparatively low in both cancerous and normal tissue from TCGA. One possible reason is that samples of TCGA were formalin-fixed paraffin-embedded, resulting impairment of RNA fragments and inaccuracy of expression levels of some genes. Overall, these findings suggested that *RRM2*, *SLC2A1*, *DDIT4*, and *VDAC2* were potential oncogene, whereas *PEBP1* and *IL33* were candidate tumor suppressor genes.

To assess the effect of these putative tumor suppressor genes/oncogenes on the development of lung adenocarcinoma, we analyzed the relation between the seven genes and tumor biological behaviors including proliferation, invasion, metastasis, cell cycle and epithelial-mesenchymal transition (EMT) using bioinformatics approaches. We firstly quantify the levels of proliferation, invasion, metastasis, cell cycle and EMT based on their respective marker genes. Then, we calculated the correlations between the hub gene and proliferation, invasion, metastasis, cell cycle and EMT. Impressively, four putative oncogenes (*RRM2*, *SLC2A1*, *DDIT4*, and *VDAC2*) were all significantly positively correlated with proliferation, invasion, metastasis, cell cycle and EMT, whereas *PEBP1* was negatively correlated with proliferation, invasion, metastasis, as well as EMT (Figure 8C).

### Assessment of the protein levels of seven genes in lung adenocarcinoma

**Figure 9:**
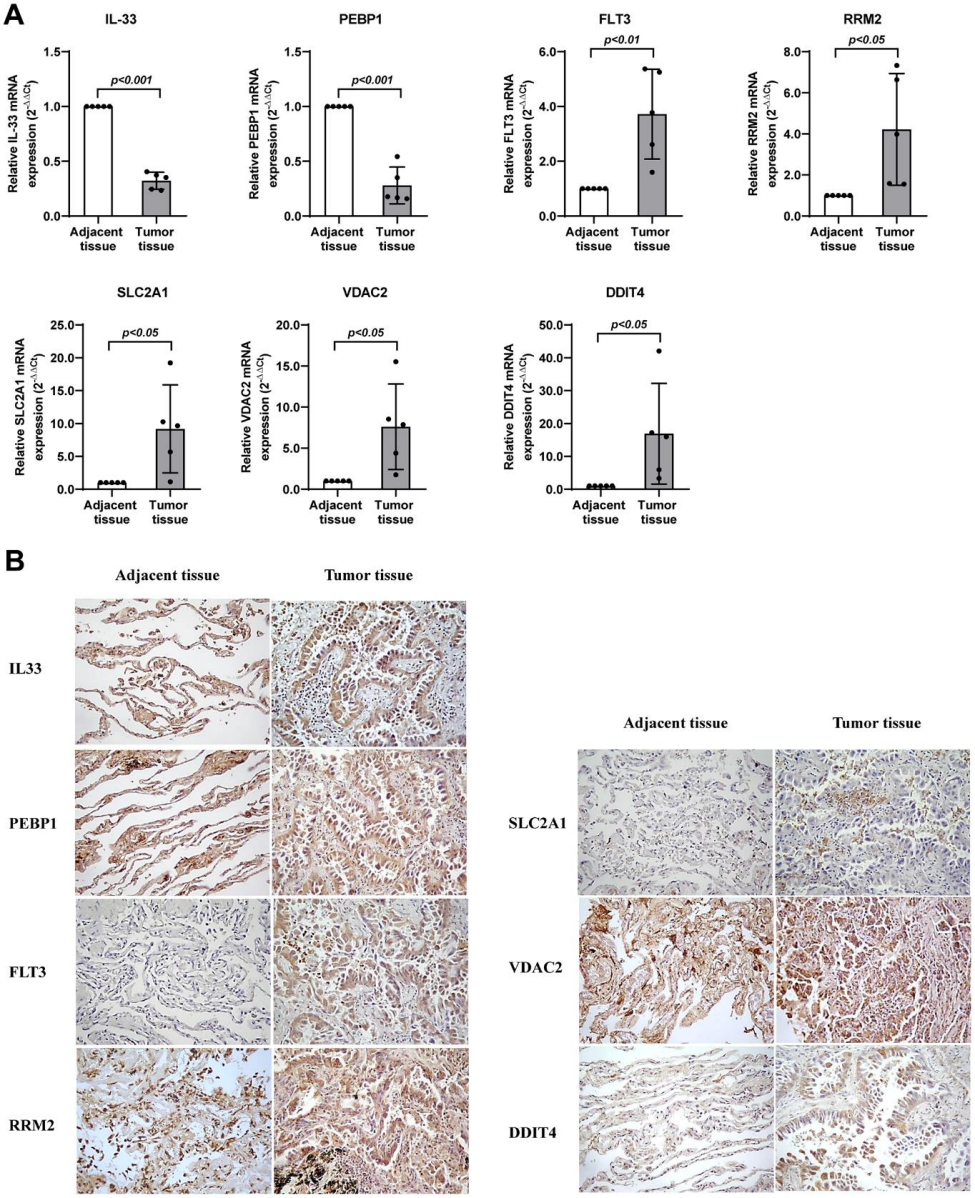
**Validation of the expression levels of seven genes in lung adenocarcinoma.** (**A**) RT-qPCR assay detected mRNA levels of the seven genes related with ferroptosis in lung adenocarcinoma. (**B**) Immunohistochemistry assay detected protein levels of the seven genes related with ferroptosis in lung adenocarcinoma. * represents *P* < 0.05, ** represents *P* < 0.01, *** represents *P* < 0.001.

We had identified the seven genes to be key genes that were linked to prognosis and tumor biological behaviors, and thus we sought to further validate their expression levels using the snap-frozen samples of lung adenocarcinoma patients from our hospital. Five pairs of tumor and adjacent normal samples were collected, and were used for the subsequent lab experiments. As we expected, *RRM2*, *SLC2A1*, *DDIT4*, and *VDAC2* were significantly upregulated transcriptionally and translationally in tumor samples (Figure 9), whereas *PEBP1* and *IL33* are significantly reduced in tumorous tissues (Figure 9).

## DISCUSSION

Recent studies showed the role of ferroptosis in suppressing tumor growth and enhancing curative effects [17], while the practical value of ferroptosis in lung adenocarcinoma is unclear [18]. Moreover, the prognosis remains challenging due to the underlying molecular heterogeneity and diverse etiology of lung adenocarcinoma [19]. Therefore, there is a critical need to develop effective prediction models [20]. Here, we constructed a new signature to foresee overall survival in lung adenocarcinoma patients. Also, we identified four candidate oncogenes, which were linked to cancer behaviors including proliferation, invasion, metastasis and EMT, and the protein levels of the candidate oncogenes were validated through qRT-PCR and immunohistochemistry.

The study revealed that *RRM2, SLC2A1, DDIT4*, and *VDAC2* were positively associated with the survival risk of lung adenocarcinoma patients. Ribonucleotide reductase regulatory subunit M2 (*RRM2*) is implied in a variety of cancers, consisting of glioma, bladder cancer, and lung cancer [21], and correlated with ferroptosis in a GSH-dependent manner [22]. *SLC2A1* is a prognostic protein for pancreatic cancer patients [23]. *SLC2A1* can suppress ferroptosis by stimulating *SLC2A1* in lung cancer [24]. *DDIT4* participates in the occurrence of tumors and affects the survival of patients [25], which adversely affects the survival of lung adenocarcinoma [26]. Furthermore, *DDIT4* can result in the pharmacodynamics inhibition of cystine-glutamate exchange [27]. Voltage dependent anion channel 2 (*VDAC2*) can inhibit iron sagging in melanoma [24, 26].

On the other hand, *PEBP1, FLT3* and *IL33* were adversely associated with the outcomes of lung adenocarcinoma patients. Phosphatidylethanolamine-binding protein 1 (*PEBP1*) can promote ferroptosis in asthma, kidney injury, and brain trauma [28], and inhibit the metastasis of tumor cells [29]. On the contrary, down-regulated *PEBP1* has been observed to link to poor prognosis [30]. *FLT3* binds proteins of the cellular iron metabolism under ferroptosis stress. *FLT3* inhibitors are considered to be effective protective agents that can inhibit the toxicity of glutamate and the production of ROS [31]. The role of *FLT3* in lung cancer is not clear now, while FLT3 ligands were considered as hematopoietic stimulators and can be employed in lung immune cell populations [32]. Here, we found that *FLT3* was linked to an improved survival, whereas its expression was elevated in the lung cancer samples. These implied that *FLT3* might serve as a tumor suppressor gene and could be upregulated in feedback with the increase of tumor malignancy; however, the specific function and molecular mechanism need to be further studied. Interleukin-33 (IL-33) is a multifunctional cytokine [33]. IL-33 can facilitate lung metastasis by regulation of the immune microenvironment [34]. Fibroblast-derived IL-33 induces breast cancer progression by changing type-2 immunity [35]. IL-33 and its receptor suppression of tumorigenicity 2 (ST2) are considered as prognostic markers for poor outcomes in gastric cancer [34].

Some ferroptosis-related gene signatures have been established in recent research and applied to lung adenocarcinoma before [7]. However, there are several aspects that highlights our signature is distinct to the previous. First, the modeling pipeline is more rigorous due to the validation of the gene signature not only in the training set but in the two independent external test sets (GSE8894 and GSE72094). In addition, the signature in this study is predictive of immunotherapeutic effects, which is supported by several indicators such as TMB and MSI and validated in the IMvigor210 cohort. Furthermore, the genes consisting the prognostic signature were further analyzed and suggested to be candidate oncogenes using the computational and laboratory experiments. In contrast to the previous study that identified prognostic gene signatures in lung adenocarcinoma, the AUC values could reach to 0.657/0.659/0.687 and 0.667/0.677/0.752 for 1, 2, and 5 years in GSE8894 or GSE72094 datasets, which are higher than those of Sun’s gene signature, with only 0.625, 0.588, and 0.593 for 2, 3, and 5 years [7]. Moreover, the qRT-PCR and immunohistochemistry results successfully validated that these mRNA and protein levels of the 7 ferroptosis-related genes are consistent with the gene signature prediction of prognosis in lung adenocarcinoma patients.

Several limitations should be noticed. First, it was a retrospective study, and all data were from the retrospective samples. Second, the model needs to be further assessed using a bigger clinical sample size. Finally, besides its good performance in distinguishing lung adenocarcinoma from normal lung, the role of 7 ferroptosis-related gene signature in distinguishing normal lung, lung nodules and small cell lung cancer also needs to be further elucidated.

In summary, our study found a ferroptosis-related signature to foresee survival and immunotherapeutic effects. The signature could be helpful to the risk stratification and cancer management. Finally, we discovered four candidate oncogenes using the computational and laboratory experiments, which could serve as potential therapeutic molecular targets.

## MATERIALS AND METHODS

### Public datasets and data processing

Data of the TCGA cohort of patients with lung adenocarcinoma were downloaded from UCSC Xena on Nov 20, 2021, including 52113 genes for 585 lung tissue, among which there existed 526 cancerous tissue and 59 normal samples, as well as the corresponding survival data. Meanwhile, data for patients with lung adenocarcinoma were also acquired from GSE8894 and GSE72094. Somatic nucleotide variation data of lung adenocarcinoma patients were acquired on UCSC Xena on Nov 20, 2021.

Gene expression data from TCGA were transformed into the TPM format and log2-transformed. GSE8894 was sequenced on Affymetrix U133 Plus 2.0 platform and normalized using GCRMA algorithm. GSE72094 was sequenced using Rosetta/Merck Human RSTA Custom Affymetrix 2.0 microarray and subjected to IRON normalization.

### Human samples

Cancerous samples and paired tumor-adjacent samples were from lung adenocarcinoma patients in the First Affiliated Hospital of Jilin University. Written informed consent was acquired and was approved by the Ethics Committee of the First Hospital of Jilin University.

### Analysis of gene expression levels

Differentially expressed genes (DEGs) were calculated using R package “edgeR”. R package “edgeR” resorts to the negative binomial distribution to explore the variable variability. DEGs were screened by |logFC| > 2 and FDR < 0.001.

### Establishment and assessment of the prognostic signature

Patients were randomly classified into the discovery and the assessment group. The univariate Cox regression and LASSO regression analysis were utilized to discovery the signature. Area under the curve (AUC), C-index, Kaplan-Meier curve were used to assess the signature.

### Quantitative reverse transcription polymerase chain reaction (qRT-PCR)

Trizol reagent (Vazyme Biotech, Nanjing, China) was used to isolate the total cellular RNA from lung adenocarcinoma and adjacent tissues. Then, the RNA was reverse transcribed to obtain the cDNA by the Prime Script RT Master Mix reagent (Takara Bio, Dalian, China). TB Green® Premix Ex Taq™ (Takara Bio, Dalian, China) and Applied Biosystems StepOnePlus real-time PCR system (Thermo Fisher Scientific, USA) were utilized to perform PCR. The *RRM2*, *IL33*, *SLC2A1*, *PEBP1*, *DDIT4*, *VDAC2*, *FLT3* mRNA levels were amplified by the following primers (Table 1), *GAPDH* was used as a reference. The entire workflow was consistent with the corresponding protocols.

**Table 1:** The sequences of primer in this study.

**Gene symbol**	**Primer sequences (5’ to 3’)**	
	**Forward**	**Reverse**
GAPDH	TTGGCTACAGCAACAGGGTG	GGGGAGATTCAGTGTGGTGG
PEBP1	CACCAGCATTTCGTGGGATG	CAGGAAATGATGCCATTCTCTGT
IL33	TGTGTTTCAAGCTGGGAAAATCC	ATCATAAGGCCAGAGCGGAG
FLT3	ACAAGTCTCCCAACTGCACA	CTCGACACCCACTGTCCAAA
RRM2	AGAGGCTACCTATGGTGAACG	TCAGTCCTCGTTTCTTGAGCC
SLC2A1	TCTGGCATCAACGCTGTCTT	AGCCAATGGTGGCATACACA
DDIT4	GGTTTGACCGCTCCACGAG	GGTAAGCCGTGTCTTCCTCC
VDAC2	GCTCAGATTGCCTGCCCTTA	TCACCAACCCAAAACCAAATCC

### Immunohistochemistry

Immunohistochemistry was carried out to verify the protein expression in this study. In brief, all tissue sections were deparaffinized and rehydrated, and then heat-mediated antigen retrieval was performed. Slides were blocked with normal goat serum, and incubated with anti-RRM2 (DF7248, Affinity, USA), anti-IL33 (YT5487, Immunoway, USA), anti-SLC2A1 (YM6583, Immunoway), anti-PEBP1 (YT4150, Immunoway), anti-DDIT4 (NBP1-77321SS, Novus, USA), anti-VDAC2 (AF5397, Affinity) and anti-FLT3 (YT6224, Immunoway) antibodies overnight (4° C). Tissue sections were then stained with HRP conjugated secondary antibody (Vector Lab, USA). The positive expression of proteins was mainly located in the cytoplasm.
